# Central projections from Johnston’s organ in the locust: Axogenesis and brain neuroarchitecture

**DOI:** 10.1007/s00427-023-00710-0

**Published:** 2023-09-11

**Authors:** George Boyan, Leslie Williams, Erica Ehrhardt

**Affiliations:** 1 Graduate School of Systemic Neuroscience, Biocenter, Ludwig-Maximilians-Universität München, Grosshadernerstrasse 2, 82152 Munich, Planegg-Martinsried, Germany; 2 Institute of Zoology, AG Ito, Universität Zu Köln, Zülpicher Str. 47B, 50674 Cologne, Germany

**Keywords:** Locust, Embryo, Johnston’s organ, Axogenesis, Central pathway, Brain

## Abstract

Johnston’s organ (Jo) acts as an antennal wind-sensitive and/or auditory organ across a spectrum of insect species and its axons universally project to the brain. In the locust, this pathway is already present at mid-embryogenesis but the process of fasciculation involved in its construction has not been investigated. Terminal projections into the fine neuropilar organization of the brain also remain unresolved, information essential not only for understanding the neural circuitry mediating Jo-mediated behavior but also for providing comparative data offering insights into its evolution. In our study here, we employ neuron-specific, axon-specific, and epithelial domain labels to show that the pathway to the brain of the locust is built in a stepwise manner during early embryogenesis as processes from Jo cell clusters in the pedicel fasciculate first with one another, and then with the two tracts constituting the pioneer axon scaffold of the antenna. A comparison of fasciculation patterns confirms that projections from cell clusters of Jo stereotypically associate with only one axon tract according to their location in the pedicellar epithelium, consistent with a topographic plan. At the molecular level, all neuronal elements of the Jo pathway to the brain express the lipocalin Lazarillo, a cell surface epitope that regulates axogenesis in the primary axon scaffold itself, and putatively during fasciculation of the Jo projections to the brain. Central projections from Jo first contact the primary axon scaffold of the deutocerebral brain at mid-embryogenesis, and in the adult traverse mechanosensory/motor neuropils similar to those in *Drosophila*. These axons then terminate among protocerebral commissures containing premotor interneurons known to regulate flight behavior.

**Supplementary Information:**

The online version contains supplementary material available at 10.1007/s00427-023-00710-0.

## Introduction

Johnston’s organ (Jo) functions as an antennal auditory organ across a spectrum of insect species (Tischner 1953; Manning 1967; Göpfert and Robert 2001a, b; Todi et al. 2004; Boekhoff-Falk 2005; Grob et al. 2021), mediates dance communication in bees (Brockmann and Robinson 2007), and as a wind-sensitive receptive system regulates flight behavior in locusts (Gewecke 1970, 1972) and *Drosophila* (Mamiya and Dickinson 2015; Patella and Wilson 2018). Afferents from Johnston’s organ have been shown to project from the antenna to the adult brain in several species including ant (Grob et al. 2021), *Drosophila* (Kamikouchi et al. 2006; Patella and Wilson 2018) and bee (Brockmann and Robinson 2007). In the bee, brain projections are organized topographically so as to conserve the spatial information encoded by the sensilla in the antenna (Ai et al. 2007).

**Fig. 1:**
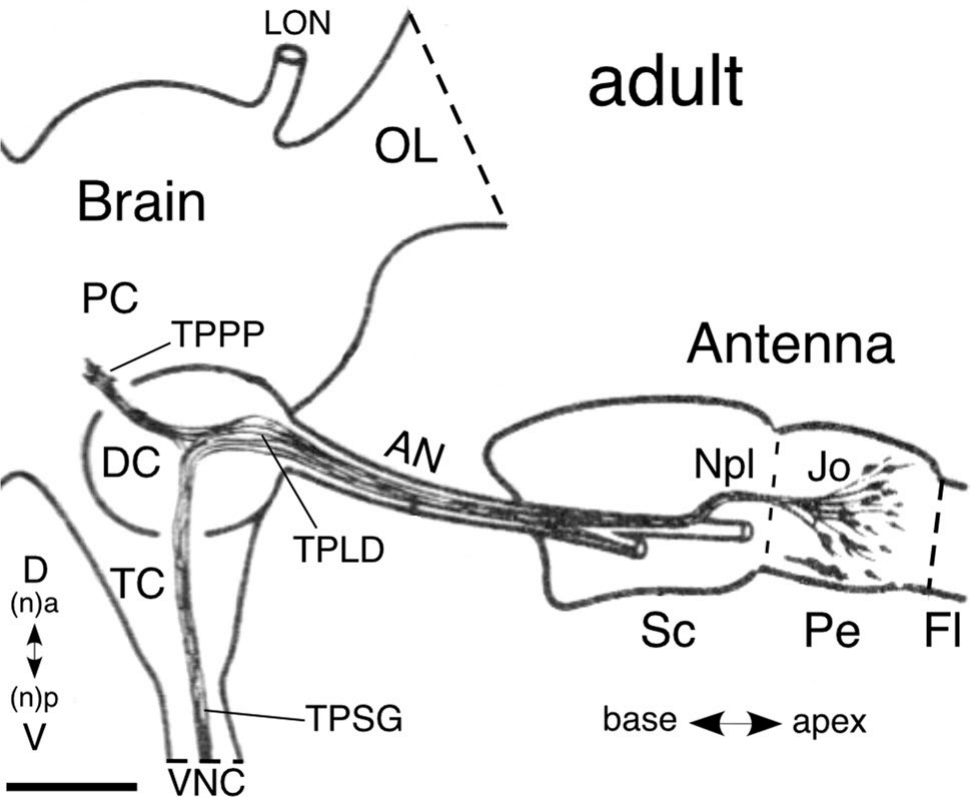
Semi-schematic diagram of the antennal base and right brain hemisphere of the adult locust seen frontally and in posterior (n, dorsal) view shows cell clusters of Johnston’s organ (Jo) in the pedicel (Pe) along with their axonal projections into the brain as revealed by cobalt backfilling (modified from Gewecke 1979 with permission of Schweizerbart Publications). Bundled axons from Jo form the Nervus pedicellaris lateralis (Npl) and project as the Tractus Pedicello-Lobus Dorsalis (TPLD) into the deutocerebrum (DC) via the antennal nerve (AN). The TPLD subsequently runs dorsal (n, anterior) to the antennal lobe and terminates as the Tractus Pedicello-Protocerebrum Posterior-medianus (TPPP) medially in the protocerebrum (PC) of the brain. A Tractus-Pedicello-ganglion Subesophagealis (TPSG) revealed by cobalt backfilling from the ventral nerve cord (VNC) descends to the subesophageal ganglion (not shown). The axons in this tract derive from unidentified cells in the pedicel. Other abbreviations: Fl, flagellum; LON, lateral ocellar nerve; OL, optic lobe; Sc, scape; TC, tritocerebrum. Body axes and equivalent neuraxes (n) are indicated. Scale bar represents 330 µm

To our knowledge, the only reports of projections from Jo into the brain of the adult locust are an observation based on Golgi histology (Williams 1972) and a cursory study using cobalt backfilling (see Fig. 1; Gewecke 1979). Both showed axons entering the deutocerebrum from the antennal nerve and terminating near the protocerebral midline but lacked information about the location of these central projections with respect to neuropilar regions (c.f. von Hadeln et al. 2018) and the commissural system (c.f. Boyan et al. 1993) of the brain. Such information would allow the circuitry mediating flight behavior to be resolved and provide comparative data offering insights into its evolution.

From a developmental perspective, neurogenesis in Johnston’s organ itself has been extensively studied in *Drosophila* (see Eberl and Boekhoff-Falk 2007; Jarman 2014; Singhania and Grueber 2014), mosquito (Schmidt 1967), moth (Keil and Steiner 1990; Keil 1997), cockroach (Blöchl and Selzer 1988), and locust (Boyan and Ehrhardt 2022), but axogenesis of the afferent pathway from Jo to the brain is less well understood. In holometabolous insects, its development commences during embryogenesis but only reaches maturity during the pupal to adult transition (see Singhania and Grueber 2014). In hemimetabolous insects, on the other hand, mechanosensory systems must be functional on hatching and we have shown that by mid-embryogenesis, the overall pattern of tracts associated with Jo in the locust antenna already strongly resembles that described for the adult (Boyan and Ehrhardt 2022). However, the process of axogenesis enabling afferents from Johnston’s organ to reach their targets in the brain has yet to be elucidated.

The locust antenna contains two major axon tracts and these are pioneered early in embryogenesis by neurons from its apical tip (Ho and Goodman 1982; Seidel and Bicker 2000; Boyan and Williams 2004; Ehrhardt et al. 2015). Axons from these pioneers project first to the deutocerebrum and then the protocerebrum where they target the primary axon scaffold of the brain (Boyan and Ehrhardt 2015). In this present study, we report that projections from cell clusters of Jo stereotypically associate with only one axon tract according to their location in the pedicellar epithelium, consistent with a topographic plan. At the molecular level, all neuronal elements of the Jo pathway to the brain express the lipocalin Lazarillo, a cell surface epitope that regulates axogenesis in the antennal axon scaffold (Ehrhardt et al. 2015). Central projections from Jo first contact the primary axon scaffold of the deutocerebral brain at mid-embryogenesis, and in the adult traverse mechanosensory/motor neuropils (von Hadeln et al. 2018) similar to those in bee, ant, and fly (Kamikouchi et al. 2006; Ai et al. 2007; Brockmann and Robinson 2007; Patella and Wilson 2018; Grob et al. 2021). In the locust, axon terminals from Jo are found among commissures containing the processes of premotor interneurons regulating flight behavior (Boyan et al. 1993).

## Materials and methods

### Animal source

Locusts (*Schistocerca gregaria)* used in this study derive from crowded colonies maintained at the Biocenter, Ludwig-Maximilians-Universität München (see Ehrhardt et al. 2015). Embryos were staged to the nearest 1% of developmental time (5% ≈ 24 h under colony conditions) according to Bentley et al. (1979). The results presented in this paper derive from experiments on over 80 embryonic and adult locusts.

### Immunolabeling

Protocols for immunolabeling with primary and secondary antibodies, microscopy, and image processing were all as previously described (see Boyan and Williams 2004, 2007; Boyan and Niederleitner 2011; Ehrhardt et al. 2015).

The following antibodies were employed:

### Primary antibodies

Anti-Horseradish peroxidase (α-HRP, polyclonal rabbit, Dianova) recognizes a neuron-specific cell surface epitope in insects (see Jan and Jan 1982).

Anti-Lazarillo (Mab 10E6, mouse, generous gift of D. Sánchez) recognizes the glycosylphosphatidylinositol (GPI)-linked cell surface lipocalin Lazarillo expressed by sensory and pioneer neurons in the locust embryo (see Sánchez et al. 1995; Ganfornina et al. 1995).

Anti-8B7 (Mab, mouse, generous gift of M. Bastiani) recognizes the Akt2 isoform of protein kinase B. In locusts, the Akt2 kinase is expressed early in development in neuroblasts and their progeny, later in axonal projections (see Seeger et al. 1993).

Anti-Lachesin (Mab 1C10, mouse, generous gift of M. Bastiani) recognizes the glycosylphosphatidylinositol (GPI)-linked cell surface molecule Lachesin belonging to the Ig superfamily (see Karlstrom et al. 1993). The expression occurs initially on all differentiating epithelial cells, but only cells involved in neurogenesis, such as precursors, continue to express the molecule later.

### Secondary antibodies

Single labeling: against α -HRP involved Alexa® 488 (goat anti-rabbit, Invitrogen) or Cy3 (goat anti-rabbit, Dianova); against either α -Lazarillo, α -8B7, or α -Lachesin involved Cy3 (goat anti-mouse, Dianova).

Double labeling: against α -HRP/ α -Lazarillo involved Alexa® 488 (goat anti-rabbit, Invitrogen) for α -HRP and Cy3 (goat anti-mouse, Dianova) for α -Lazarillo; against α -HRP/ α -Lachesin involved Cy5 (donkey anti-goat, Dianova) for α -HRP/Alexa® 488 (donkey anti-mouse, Invitrogen) for α -Lachesin; against α -HRP/ α -8B7 involved Alexa® 488 (goat anti-rabbit, Invitrogen) for α -HRP/ Cy3 (goat anti-mouse, Dianova) for α -8B7.

Controls for the specificity of all secondary antibodies were (a) the lack of a staining pattern in the absence of the primary antibody and (b), in all cases, a staining pattern consistent with previously published data (see above).

### Histology

#### Golgi histology

This employs silver impregnation to reveal neuronal morphology. The protocol for both adult and embryonic locusts basically followed the Golgi-Kopsch method of Colonnier as described in Williams (1972).

#### Bielschowsky histology

This employs silver impregnation to highlight mainly axonal membranes and/or neurofilaments. The protocol used is described in Boyan et al. (1993).

Embryonic preparations were sectioned at 14–16 µm thickness for microscopy, adult preparations at 25–100 µm thickness.

### Nomenclature

This study applies body axes and the equivalent neuraxes (denoted by the affix "n") to brain anatomy. The nomenclature used to describe brain neuroarchitecture follows Boyan et al. (1993) for the commissural system, and Ito et al. (2014) and von Hadeln et al. (2018) for tracts and neuropils. The nomenclature of Gewecke (1979) is maintained for the projections of Jo afferents in the locust brain as these are not described elsewhere. Anatomical abbreviations are explained directly in the relevant figure legend.

## Results

### Background

Gewecke (1979) showed that axons from cell clusters of the Jo merge to form the Nervus pedicellaris lateralis (Npl) and then project into the deutocerebrum from the antennal nerve as the so-called Tractus Pedicello-Lobus Dorsalis (TPLD) (Fig. 1). The TPLD subsequently runs dorsally (n, anterior) and posteriorly (n, dorsal) to the antennal lobe and terminates as the Tractus Pedicello-Protocerebrum Posterior-medianus (TPPP) medially in the protocerebral brain. We confirm these pioneering findings but are unable to replicate Gewecke’s finding of a Tractus-Pedicello-ganglion Subesophagealis (TPSG) that splits from the TPLD itself and descends in the ventral nerve cord (VNC) to the subesophageal ganglion. The axons in this tract were revealed by cobalt backfilling from the VNC and purportedly derive from cells in the pedicel, but until their identity is clarified, we focus here on the protocerebral terminations in the TPPP tract which allow a direct comparison to be made with those in ant (Grob et al. 2021), *Drosophila* (Kamikouchi et al. 2006; Patella and Wilson 2018), and bee (Brockmann and Robinson 2007).

### Axogenesis and fasciculation with pioneer antennal tracts

**Fig. 2:**
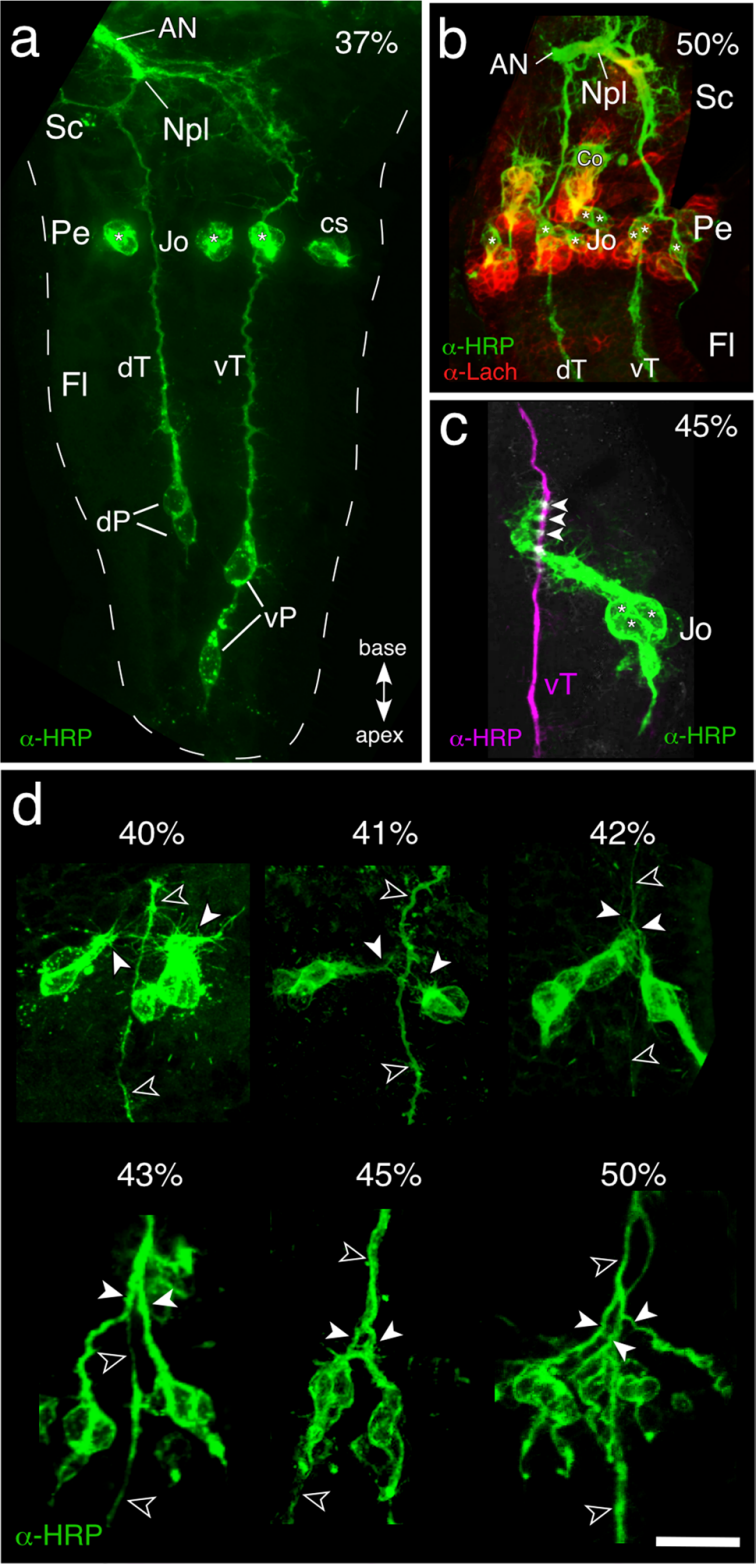
The primary axon scaffold of the antenna. **a** Confocal image following immunolabeling with α -HRP at 37% of embryogenesis shows HRP-positive sibling dorsal (dP) and ventral (vP) pioneer cells in the apical flagellum (Fl) have, respectively, generated the initial antennal tracts (dT, vT). These tracts merge to form the Nervus pedicellaris lateralis (Npl, see Fig. 1 for adult) in the scape (Sc) and will project as part of the antennal nerve (AN) to the brain. Cell clusters of Jo (white stars) in the pedicel (Pe) are also labeled but have yet to generate filopodia. Antennal coordinates shown apply throughout. Other abbreviation: cs, campaniform sensillae. Modified from Boyan and Ehrhardt (2019). **b** Confocal image following double-immunolabeling with α -HRP (green) and epithelial cell-specific Lachesin (α -Lach, red) at 50% of embryogenesis shows HRP-positive axons from Jo cell clusters (white stars) in the pedicellar domain have projected onto the primary antennal tracts (dT, vT) and then merge at the antennal base to form the Npl which joins the AN. Other abbreviation: Co, chordotonal organ. **c** Confocal image at 45% of embryogenesis shows HRP-positive cells (white stars) of a Jo cluster whose processes (green) fasciculate with the HRP-positive vT tract (magenta) at three parallel sites (white arrowheads). **d** Confocal images at successive embryonic ages following α -HRP-labeling document progressive fasciculation of cell clusters from Jo with a pioneer ventral tract (vT, open white arrowheads throughout). At 40%, initial filopodia from the growth cones (white arrowheads) of two clusters appear but are not yet clearly directed towards the vT; at 41%, filopodia (white arrowheads) from both clusters are now clearly directed towards the vT, which also sprouts multiple filopodia; at 42%, filopodia (white arrowheads) from both cell clusters extend anteriorly and fasciculate with the vT; at 45%, processes (white arrowheads) from both cell clusters clearly merge with one another and with the vT; at 50%, processes (white arrowheads) from several cell clusters fasciculate with one another and with the vT. Scale bar represents 25 µm in a, 55 µm in b, 30 µm in c, and 35 µm in d

In hemimetabolous insects, mechanosensory structures such as Johnston’s organ (Jo) must be functional on hatching and so develop during embryogenesis (see Anderson 1973; Boyan and Ehrhardt 2022). We therefore began our investigation of axogenesis in the pathway from Jo to the brain at early embryonic stages. Immunolabeling of a neuron-specific cell surface epitope recognized by the antibody against horseradish peroxidase (α -HRP) shows that by 37% of embryogenesis (Fig. 2a), HRP-positive dorsal and ventral pioneer cells in the apical flagellum have generated two respective tracts that then merge at the antennal base and project within the antennal nerve towards the brain. The pathway from Jo itself to the brain has still to form as the cell clusters of Jo have yet to undergo axogenesis. However, double-immunolabeling with α -HRP and anti-Lachesin (α -Lach) (Fig. 2b) confirms that at 50% of embryogenesis, a Nervus pedicellaris lateralis (Npl; c.f. Fig. 1) comprising axons both from the Jo cell clusters in the pedicellar domain and the pioneer tracts is present, implying that construction of the afferent pathway from Jo to the brain must occur beforehand. Significantly, outgrowing axons from a Jo cell cluster are also clearly seen to fasciculate with a pioneer antennal tract (Fig. 2c) suggesting that these might act as a guidance system for Jo axons tracking to the brain.

A time series following immunolabeling with α -HRP (Fig. 2d) reveals that fasciculation between the axons from Jo cell clusters and the primary antennal tracts develops in a stepwise manner. At 40% of embryogenesis, the axonal growth cones of Jo cells generate initial filopodia but these are not yet specifically directed towards a pioneering antennal tract. At 41%, filopodia from cells in two clusters are now clearly directed towards an antennal tract that also sprouts multiple filopodia in this region. Filopodia from Jo cell clusters then extend basally in the antenna (42%), fasciculate with one another (43%), and also project onto a pioneer antennal tract (45%). At 50%, axons from several cell clusters can be seen projecting together onto a single pioneering tract, confirming our hypothesis.

### Fasciculation is topographic

**Fig. 3:**
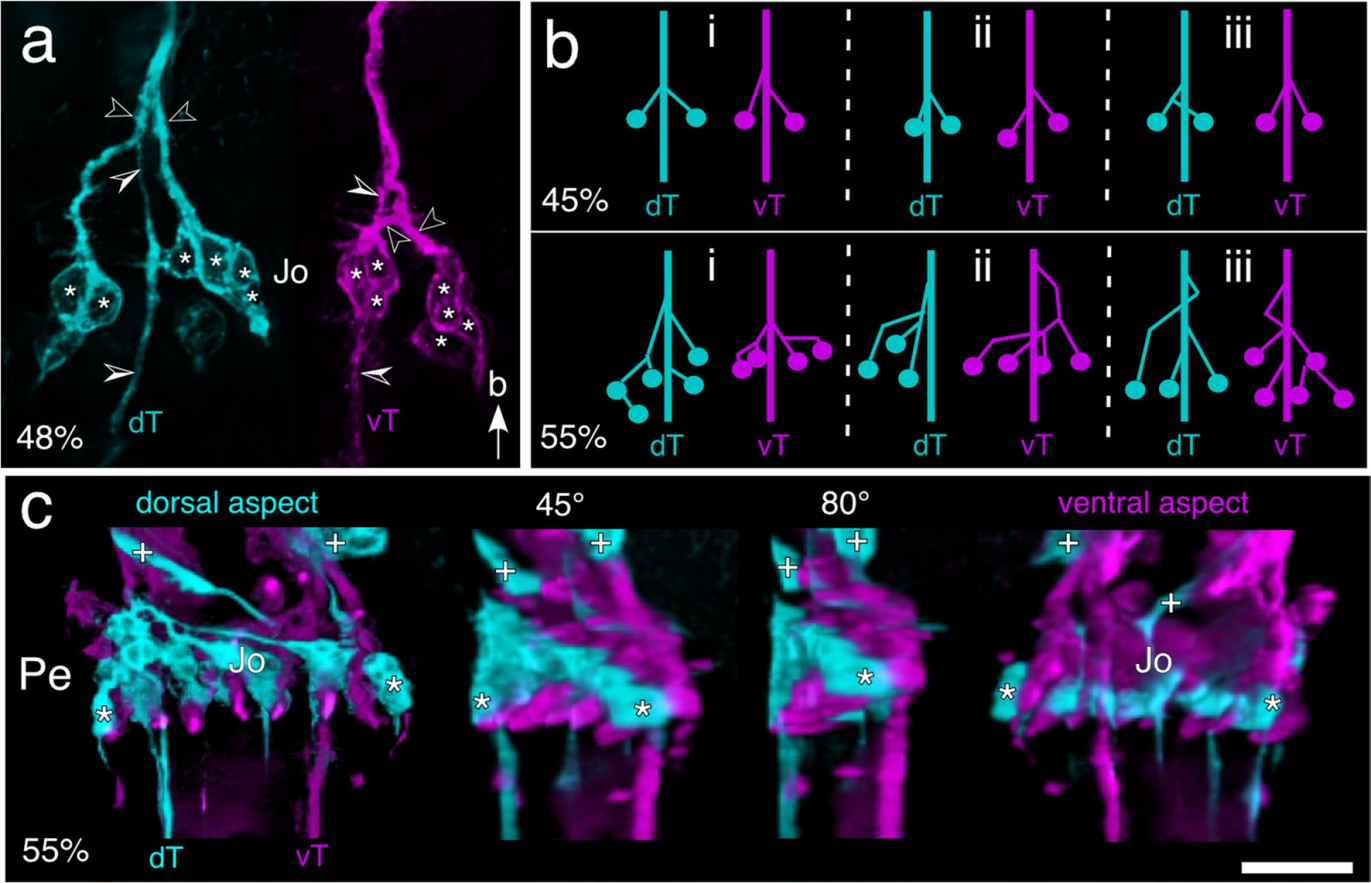
Cell clusters of Jo fasciculate selectively with pioneer axon tracts of the antenna. **a** Confocal image following α -HRP immunolabeling at 48% of embryogenesis shows HRP-positive cells (white stars) of Jo directing axonal processes (open white arrowheads) selectively onto either the dT (cyan, left) or vT (magenta, right) axon tracts (open/white arrowheads) of the antenna. Arrow points to antennal base and applies throughout. **b** Schematic summarizes fasciculation patterns between cell clusters of Jo and the two axon tracts of the antenna (dT, cyan; vT, magenta) in three repeat preparations (i–iii) at 45% (upper row) and 55% (lower row) of embryogenesis. Note that cell clusters remain consistently associated with only one or the other axon tract. **c** 3D confocal images show dorsal (cyan) and ventral (magenta) HRP-positive cell clusters in the pedicel (Pe) of the antenna at 55% of embryogenesis. The antenna is viewed first from dorsal and then rotated in two steps (45°, 80°) to fully ventral. Projections from cell clusters of Jo associate with either the dorsal (dT, cyan) or vental (vT, magenta) antennal tracts. Chordotonal (white crosses) and campaniform (white stars) cell clusters are also imaged. Scale bar represents 35 µm in a and 60 µm in c

We next examined whether projections from Jo cell clusters fasciculate selectively with a given pioneer tract according to their location (dorsal/ventral) in the pedicellar domain. Evidence for this is provided by labeling with α -HRP which shows (Fig. 3a) dorsal and ventral cell clusters each clearly fasciculating with separate pioneer tracts. Repeat preparations at 45% (Fig. 3b, upper row) and 55% (Fig. 3b, lower row) of embryogenesis confirm this pattern of selective fasciculation. A series of 3D confocal images in which the antenna is rotated in a stepwise manner from dorsal to ventral at 55% of embryogenesis (Fig. 3c) demonstrates the topographic projection pattern of axons from the six dorsal and six ventral cell clusters in the pedicel to their respective pioneer antennal tracts.

### Expression of a regulatory molecule during axogenesis

As elsewhere in the insect nervous system (see Dickson 2002), axogenesis of the Jo pathway to the brain is likely to involve a hierarchy of regulatory molecules among which are the GPI-linked cell surface lipocalins such as Lazarillo (see Ganfornina et al. 1995; Sánchez et al. 1995). We have previously shown (Suppl. Figure 1) that immunoblocking the Lazarillo epitope in whole embryo culture dysregulates axogenesis in the pioneer tracts of the locust antenna, consistent with findings in the central nervous system (Ganfornina et al. 1995; Sánchez et al. 1995).

**Fig. 4:**
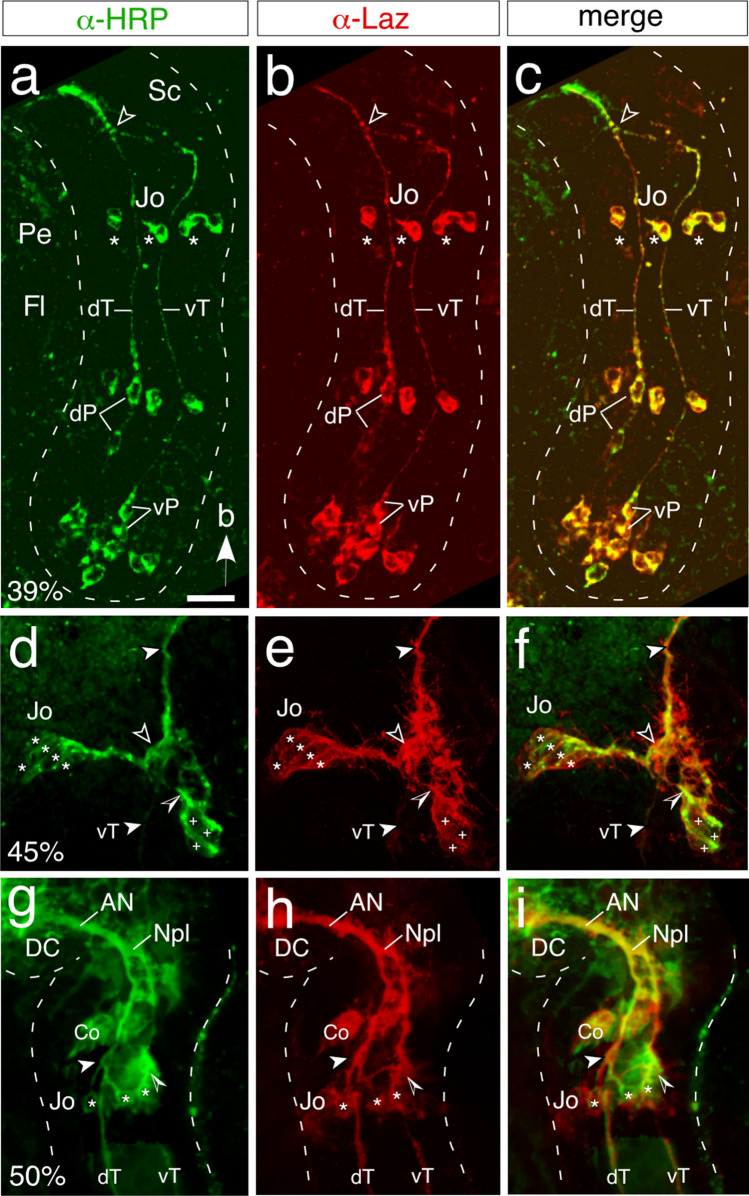
Common cell surface epitopes expressed by cell clusters of Johnston’s organ and by pioneers of the initial axon scaffold of the antenna to the brain. Panels show confocal images following double-immunolabeling with α -HRP (green) and against the GPI-linked cell surface guidance molecule Lazarillo (α -Laz, red), co-labeling (merge, yellow). **a–c** 39% of embryogenesis: co-labeling (α -HRP, a; α -Laz, b; merge, c) is evident in ventral and dorsal pioneer neurons (vP, dP) of the apical flagellum (Fl) and their axons (vT, dT) forming the primary antennal tracts. These merge to form the Npl (open white arrowhead) in the scape (Sc) en route to the brain. Cell clusters (white stars) of Johnston’s organ (Jo) in the pedicel (Pe) are co-labeled but have yet to generate axons. Arrow in panel a points to antennal base throughout. **d–f** 45% of embryogenesis: co-labeling (α -HRP, d; α -Laz, e; merge, f) is evident in two cell clusters (white stars, white crosses) of the Jo whose processes fasciculate (open white arrowhead, open/white arrowhead) with the co-labeled vT (white arrowheads). **g–i** 50% of embryogenesis: co-labeling (α -HRP, g; α -Laz, h; merge, i) is evident in cells (white stars) from a Jo cell cluster whose axon processes (white arrowheads, open/white arrowheads) fasciculate selectively with the vT or dT (c.f. Figure 2). These tracts merge at the antennal base to form the Nervus pedicellaris lateralis (Npl) and enter the deutocerebrum (DC) via the antennal nerve (AN, also co-labeled). Further abbreviation: Co, chordotonal organ. Scale bar represents 50 µm in a–c, 30 µm in d–f, and 40 µm in g–i

Expression of the Lazarillo cell surface epitope in the Jo pathway to the brain was examined by double-immunolabeling with α -Lazarillo and α -HRP at progressive developmental stages (Fig. 4). At 39% of embryogenesis (Fig. 4a–c), pioneer neurons, the primary axon tracts, and cell clusters of Jo are HRP/Lazarillo-positive. The latter have yet to generate axon processes at this early stage (c.f. Fig. 2), but by 45% (Fig. 4d–f) cells of two ventral Jo clusters can be seen to extend co-labeled axon processes that fasciculate with one another and then target the co-labeled ventral tract. Subsequently (50%, Fig. 4g–i), the Lazarillo epitope is expressed in the complete pathway involving: (i) axon processes originating from Jo cell clusters, (ii) the primary ventral and dorsal tracts with which they fasciculate, (iii) the Nervus pedicellaris lateralis containing axons from both Jo and the primary antennal tracts, and (iv) the antennal nerve to the brain. We speculate that the ubiquitous expression we report here signals a wider regulatory role for Lazarillo in axogenesis of the complete pathway from Jo to the brain.

### Projections from Johnston’s organ in the brain

#### Contact with the primary axon scaffold of the brain

Axons in the pioneer antennal tracts have been shown to enter the deutocerebrum of the brain from the antennal nerve at mid-embryogenesis before then targeting the primary axon scaffold of the protocerebrum (Boyan and Ehrhardt 2015). Given the selective fasciculation between axons from Jo and these pioneer tracts within the antenna itself (see Figs. 2 and 3), it seemed plausible that projections from Jo also follow this pre-established pathway into the central brain.

**Fig. 5:**
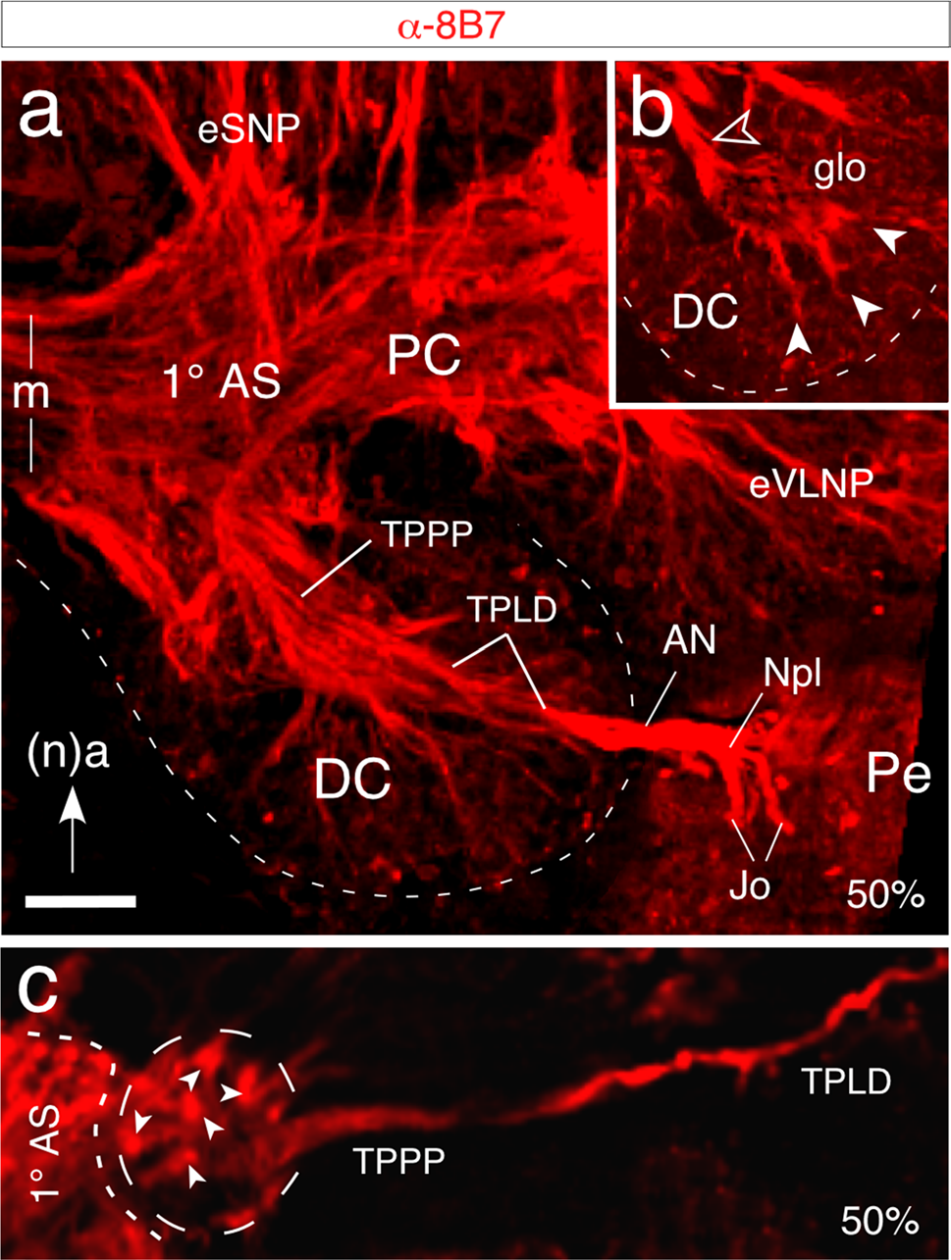
Axon projections from cell clusters of Johnston’s organ project onto the primary axon scaffold of the brain. **a** Confocal image of a longitudinal section through the antennal base and right brain hemisphere at 50% of embryogenesis following immunolabeling against axon-specific 8B7. Arrow points to (neuraxis) anterior. Axons from cell clusters of Jo form the Nervus pedicellaris lateralis (Npl) and then enter the deutocerebrum (DC) from the antennal nerve (AN) via the Tractus Pedicello-Lobus Dorsalis (TPLD, c.f. Figure 1 for adult). Jo axons in the TPLD first contact the primary axon scaffold (1° AS) of the brain in the dorsal mechanosensory region of the DC and so bypass the more ventral (body axis, anterior) glomerular region (**b**, glom) where projections from olfactory afferents (white arrowheads) terminate. Axons (open white arrowhead) from interneurons projecting to the mushroom bodies are also labeled (c.f. Strausfeld 2009). **c** Confocal image following labeling with α -8B7 at 50% of embryogenesis shows axons from Jo in the TPLD projecting via the TPPP to contact the primary axon scaffold (1° AS, short white dashes) of the brain. The terminal region (outlined, long white dashes) has the form of a dense arborization characterized by distinct blebs (white arrowheads). Other abbreviations: m, brain midline; eSNP, embryonic superior neuropils; eVLNP, embryonic ventrolateral neuropils. Scale bar represents 110 µm in a, b and 40 µm in c

We first visualized the central projections from Jo along with the axon scaffold they target in the embryonic brain by immunolabeling with axon-specific α -8B7. A longitudinal section through the antennal base and right brain hemisphere at 50% of embryogenesis (Fig. 5a) shows 8B7-labeled axons from cell clusters of Jo merging to form the Nervus pedicellaris lateralis (Npl) and then entering the deutocerebrum from the antennal nerve as the Tractus Pedicello-Lobus Dorsalis (TPLD) (c.f. Fig. 1 for the adult brain). Axons in the TPLD can be seen to contact the primary axon scaffold of the brain in the mechanosensory region of the deutocerebrum. This lies posterior (n, dorsal) to the more glomerular region (Fig. 5b) containing the projections of olfactory antennal afferents (see Williams 1972; von Hadeln et al. 2018) as well as the axons of olfactory interneurons that project to the mushroom bodies via the medial antennal lobe tract (see Ito et al. 2014; von Hadeln et al. 2018).

A further embryonic (50%) preparation (Fig. 5c) reveals that after entering the deutocerebrum, the 8B7-positive Jo axons in the TPLD project further antero-medially (according to the neuraxis) in the TPPP tract and terminate in the protocerebrum as a dense aborization characterized by distinct blebs.

## Axon tracts from Johnston’s organ and the neuroarchitecture of the brain

We then proceeded to examine the terminal projections from Johnston’s organ and potential brain neurons they might target, first using the well-established Golgi histological method (see Strausfeld 1976).

**Fig. 6:**
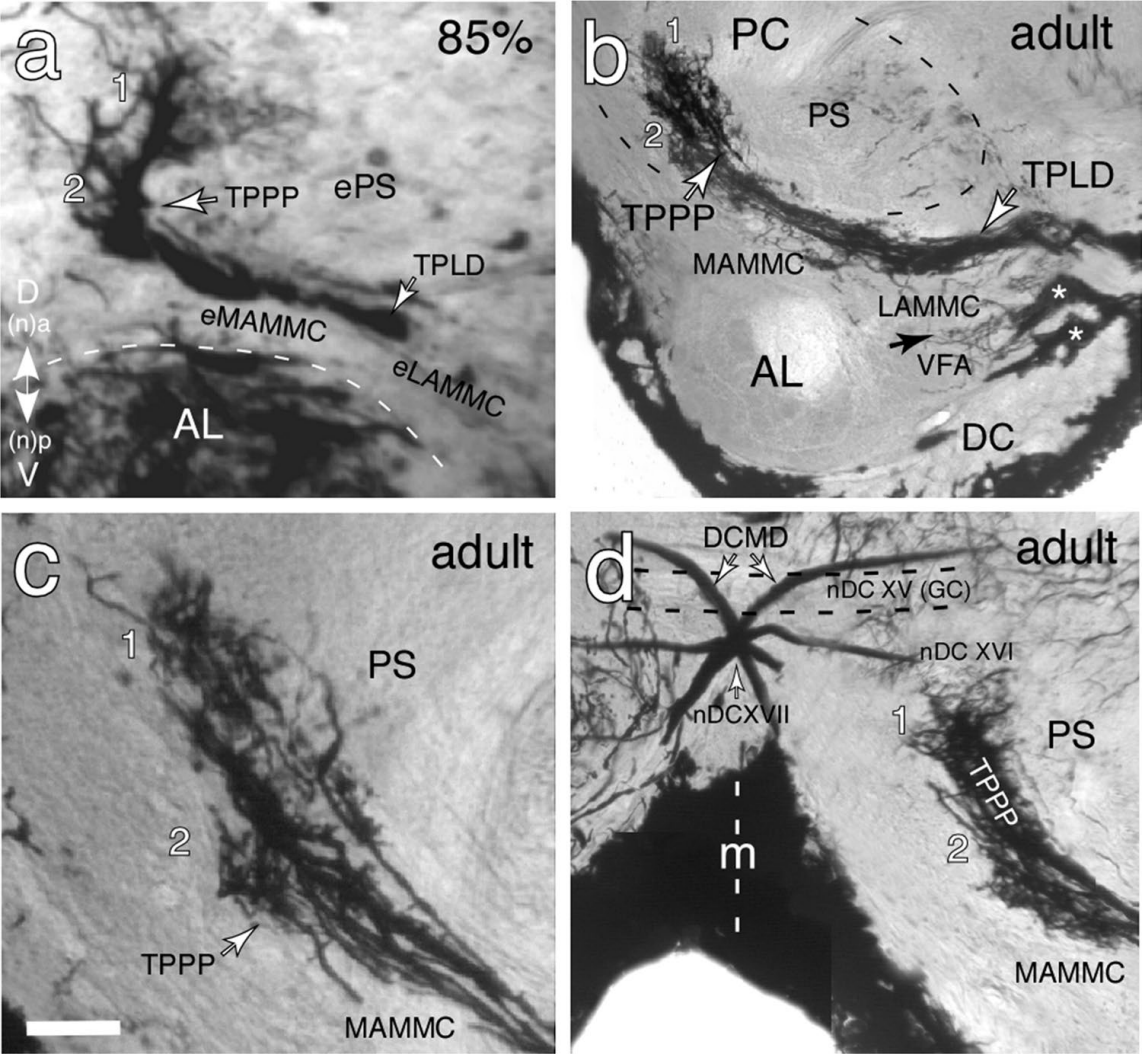
Golgi histology reveals the cerebral projections of antennal tracts containing axons from Johnston’s organ (Jo). **a** Photomicrograph (Nomarski optics) of a longitudinal section through the right brain hemisphere at 85% of embryogenesis shows axon processes from the Jo in the Tractus Pedicello-Lobus Dorsalis (TPLD) entering the deutocerebrum (DC) and projecting medially and dorsal (n, anterior) of the antennal lobe (AL, dashed white), traversing the embryonic lateral and medial antennal mechanosensory motor centers (eLAMMC, eMAMMC). Axons then turn further dorsally (n, anteriorly) in the Tractus Pedicello-Protocerebrum Posterior-medianus (TPPP) to terminate as two sub-fields (1, 2) in the mechanosensory neuropil of the embryonic posterior slope (ePS) in the protocerebrum (PC). Axes shown apply throughout. **b** Photomicrograph of a longitudinal section of the adult brain shows axon projections from Jo projecting into the DC from the TPLD and then transiting the lateral and medial antennal mechanosensory motor centers (LAMMC, MAMMC) as the TPPP dorsal (n, anterior) to the antennal lobe (AL). Axons terminate medially in the posterior slope (PS, approximate extent dashed black) of the protocerebrum. Tracts from other antennal afferents (white stars) lie further ventral (n, posterior) to the TPLD and possibly project (black arrow) into the VFA (ventral area of flagellar afferents). **c** Higher power photomicrograph from a further adult preparation shows protocerebral axon projections from Jo in the TPPP traversing the MAMMC and terminating as two sub-fields (1, 2) in the PS. **d** Photomicrograph of a longitudinal section of the adult brain shows axon projections from Jo in the PS and terminating in the region of commissural tracts (nDC XVI, XVII) containing axons of identified premotor interneurons (see Fig. 7). m, brain midline dashed white. Scale bar: represents 35 µm in a, 70 µm in b, 35 µm in c, and 65 µm in d

A longitudinal section of the right brain hemisphere at 85% of embryogenesis following Golgi histology (Fig. 6a) viewed from a posterior (n, dorsal) aspect shows the Tractus Pedicello-Lobus Dorsalis (TPLD) containing tightly bundled axon processes from the Jo in the deutocerebrum and projecting medially within the embryonic posterior slope (ePS), dorsal (n, anterior) to the developing lateral antennal mechanosensory and motor center (eLAMMC), and the antennal lobe. At the level of the embryonic medial antennal mechanosensory and motor center (eMAMMC) the axons then turn dorsally (n, anteriorly) and terminate as a dense arborization termed the Tractus Pedicello-Protocerebrum Posterior-medianus (TPPP) bordering the embryonic vest (not in view here). These protocerebral arborizations comprise two putative sub-fields and at this embryonic stage (85%) already bear a very similar overall morphology to those of the adult brain (Fig. 6b) where two sub-fields are again present. Tracts from other antennal afferents entering the deutocerebrum via the antennal nerve lie further ventral (n, posterior) to the TPLD and speculatively contribute to the ventral area of flagellar afferents (VFA).

**Fig. 7:**
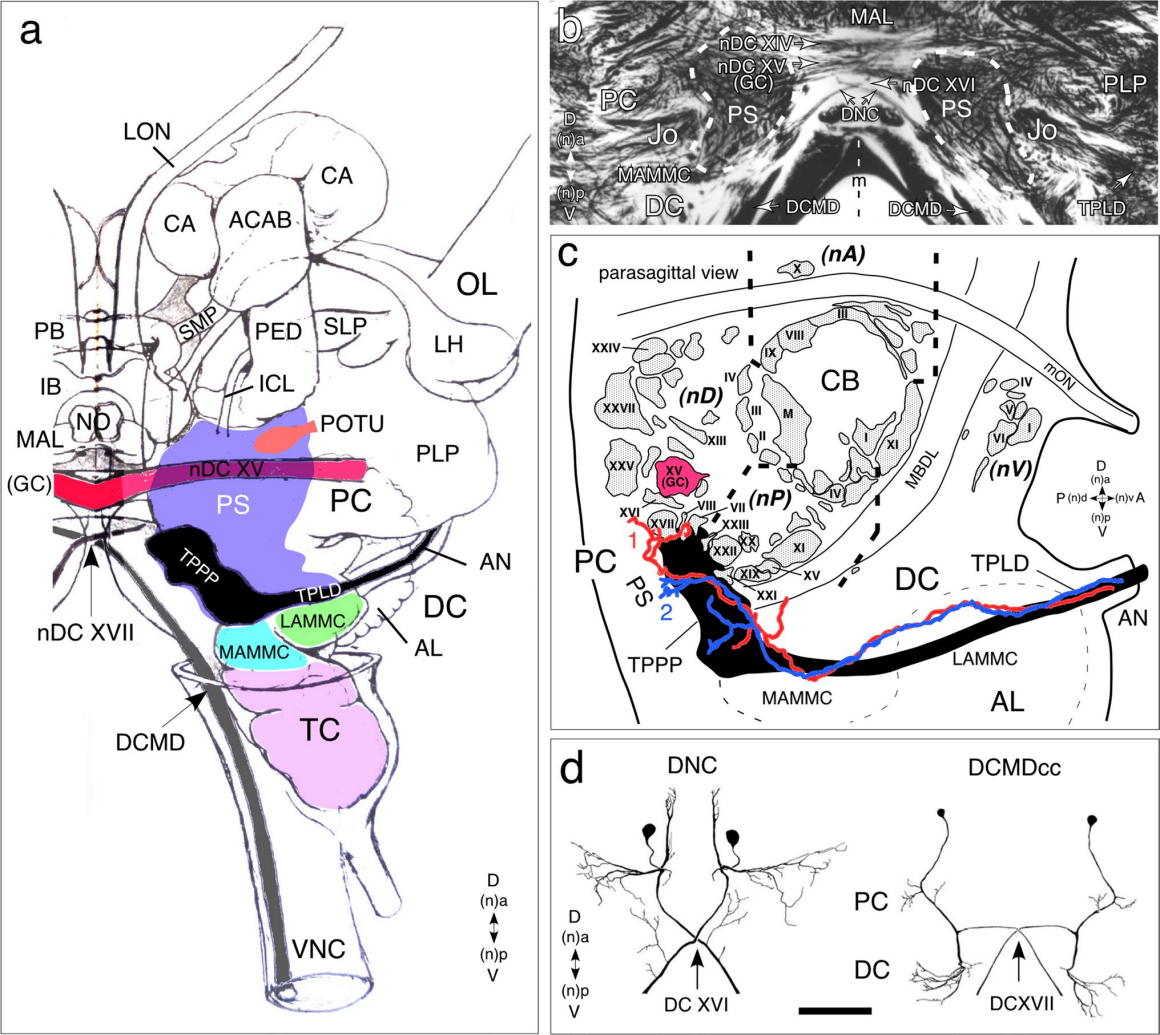
**a **Schematic reconstruction (not to scale) of the right cerebral hemisphere of the adult locust presented from posterior (n, dorsal) shows the projection of axons from Johnston’s organ (Jo, black) with respect to major neuropilar regions (nomenclature after Ito et al. 2014; von Hadeln et al. 2018) and the commissural system (nomenclature after Boyan et al. 1993). For detailed explanation, see main text. Body axes (A anterior, D dorsal, P posterior, V, ventral) and equivalent neuraxes (n) are indicated for each panel. Abbreviations: ACAB, accessory calx bulb; AL, antennal lobe; AN, antennal nerve; CA, calyx; DC, deutocerebrum; DCMD, axon of the descending contralateral movement detector; GC, great commissure (= nDC XV); IB, inferior bridge; ICL, inferior clamp; LAMMC, lateral antennal mechanosensory and motor cente; LH, lateral horn; LON, lateral ocellar nerve; MAL, medial accessory lobe; MAMMC, medial antennal mechanosensory and motor center; nDC, dorsal commissure; NO, noduli; OL, optic lobe; PB, protocerebral bridge; PC, protocerebrum; PED, pedunculus; PLP, posterior lateral protocerebrum; POTU, posterior optic tubercle; PS, posterior slope; SLP, superior lateral protocerebrum; SMP, superior medial protocerebrum; TC, tritocerebrum; TPLD, Tractus Pedicello-Lobus Dorsalis; TPPP, Tractus Pedicello-Protocerebrum Posterior-medianus; VNC, ventral nerve cord. **b** Photomicrograph of a longitudinal section of the adult brain following Bielschowsky histology shows axons from Jo of each antenna projecting symmetrically into each brain hemisphere via the TPLD tract, traversing the medial antennal mechanosensory and motor center (MAMMC) and extending as the TPPP tract to the posterior slope (PS, approximate extent dashed white). Terminals (not in section) lie in the region of dorsal commissure XVI (containing the axons of the bilateral DNC neurons (see **c**, **d**)). **c** Semi-schematic reconstruction (not to scale) from serial sections shows the organization of commissural tracts (shaded) of the adult midbrain in parasagittal view. Commissures are grouped topologically clockwise (refer to axis designations) around the central body (CB). Only selected commissural tracts are named here (see Boyan et al. 1993 for complete labeling). Computer autotracings of two Golgi-stained axons (red, blue) from the Jo have been superimposed onto the generalized path (shaded black) of the TPLD and TPPP in the brain. Both axons enter the brain ventrally via the antennal nerve (AN), traverse the LAMMC and MAMMC of deutocerebrum (DC) in the Tractus Pedicello-Lobus Dorsalis (TPLD), then turn dorsally (n, anteriorly) to terminate as two sub-fields (1, 2) within the Tractus Pedicello-Protocerebrum Posterior-medianus (TPPP) in the PS. The most anterior sub-field (1, red) lies among dorsal commissures VII, VIII, XVI, and XVII. Other abbreviations: MBDL, median bundle; mON, median ocellar nerve. **d** Reconstructions from serial sections of two identified premotor interneurons (DNC, DCMDcc) of the adult locust brain following cobalt backfilling from their axons in the ventral nerve cord (modified from Boyan et al. 1993). Both neurons have a symmetrical homolog in the other brain hemisphere, a cell body in the PC, projections in PC and DC, and an axon which crosses the brain midline in dorsal commissure XVI or XVII near the TPPP tract (c.f. Fig. 6d) to then descend to thoracic motor regions. Scale bar represents 150 µm in a, 300 µm for neuron DNC, and 200 µm for neuron DCMDcc in c

Axon counts in five preparations following Golgi histology reveal the TPPP to comprise 12, 12, 12, 10, 11 stained axons varying in diameter from 2 to 4 µm. At higher resolution (Fig. 6c), the prominent terminal blebs visible after 8B7 immunolabeling (c.f. Figure 5c) are again evident. We propose that these terminals glance the MAMMC, lie within the PS, and border the more medial VES. In favorable preparations (Fig. 6d), the Golgi method reveals axon projections from Jo terminating in two sub-fields of the TPPP and extending towards the commissural system (nDC XV (= GC), nDC XVI, nDC XVII) of the central brain. Also, stained are the protocerebral processes of identified premotor interneurons such as the bilateral descending contralateral movement detector (DCMD) neurons whose axons cross the brain midline in nDC XVII (c.f. Fig. 7 below) and then project to thoracic motor centers.

To definitively place the axon projections from Jo within the neuroarchitecture of the adult locust brain, we employed the map of brain neuropils provided by Ito et al. (2014) and von Hadeln et al. (2018). Our schematic reconstruction (Fig. 7a) shows Jo axons projecting as the TPLD into the deutocerebrum via the antennal nerve, running medially through the dorsal (n, anterior) regions of LAMMC and MAMMC and terminating as the TPPP in the posterior slope (PS) of the protocerebrum.

Sectioning following Bielschowsky histology allows the Jo axons together with the surrounding neuropilar regions and commissural tracts of the adult brain using published maps (Boyan et al. 1993; Ito et al. 2014; von Hadeln et al. 2018) to be visualized (Fig. 7b). Bundled axons from Jo of each antenna project symmetrically into the neuropil of each deutocerebral hemisphere via the TPLD tract. These axons traverse the LAMMC and MAMMC regions and run to the posterior slope (PS) near nDC XVI and nDC XVII of the protocerebrum. Repeat preparations following Bielschowsky histology consistently reveal 12 axons in this tract.

In order to analyze the central projections from the adult Jo at the single cell level, we reverted to Golgi histology, generated computer-based autotracings from two individual stained axons, and superimposed these onto the TPLD and TPPP tracts within our commissural map (Fig. 7c). Both stained Jo axons can be seen to closely follow the overall projection through LAMMC and MAMMC towards the PS (c.f. Fig. 7a). The axons terminate as two sub-fields, the most dorsal (n, anterior) field lies adjacent to commissures nDC XVI and nDC XVII containing the axons of identified multimodal premotor interneurons DNC, DCMDcc (Fig. 7d) and DCMD (c.f. Fig. 6d).

## Discussion

### Axogenesis of the pathway to the brain

Our study shows that in keeping with its hemimetabolous lifestyle, the afferent pathway from Johnston’s organ (Jo) to the brain of the locust *Schistocerca gregaria* is constructed during embryogenesis and that the primary axon scaffold of the antenna pioneered earlier by the dorsal and ventral apical pioneers (Fig. 2a) plays a key role in the process. Axogenesis commences in Jo at around 40% of embryogenesis and proceeds in a stepwise manner as the growth cones from neurons of any given cell cluster fasciculate first with one another, and then with one of the pioneer tracts (Fig. 2d). Fasciculation is selective in that cell clusters located dorsally in the pedicellar epithelial domain target the dorsal pioneer tract, and ventrally located clusters the ventral pioneer tract (Fig. 3). This topographic pattern ensures that the spatial information encoded by the sensilla from these respective cell clusters is conserved in their projections at least to the antennal base.

At the molecular level, all neuronal elements of the afferent pathway from Jo to the brain express Lazarillo (Fig. 4), one of the GPI-linked cell surface lipocalins known to regulate pioneer growth cone guidance in the locust nervous system (Chang et al. 1992; Ganfornina et al. 1995; Sánchez et al. 1995; Goodman 1996). We have shown that antibody blocking of the Lazarillo epitope alone is sufficient to dysregulate fasciculation of pioneer axons in the early antenna itself (Suppl. Figure 1; Ehrhardt et al. 2015) and speculate that Lazarillo also plays a role in regulating the fasciculation of more downstream elements in the pathway from Jo to the brain.

### Central projections

#### The embryonic brain

Although the axons from cell clusters of Johnston’s organ project selectively to either the ventral or dorsal pioneer tracts (Fig. 3), these merge at the antennal base to form the Nervus pedicellaris lateralis (Figs. 1, 2a, b, 4g–i) and so represent a single projection into the antennal nerve. In order to then preserve the topography encoded peripherally, the axons from Jo would have to maintain stereotypic locations in the antennal nerve and subsequent brain tracts. This topographic morphology is present for the axons from premotor brain neurons descending in the ventral nerve cord (Fig. 7c; c.f. Williams 1975) but has not yet been demonstrated for the afferent pathway from Jo to the locust brain. Selective labeling of dorsal and ventral clusters via combined immunolabeling and intracellular dye injection as previously used to identify pioneers and nerve tract associated cells in the embryonic antennal nervous system (Boyan and Williams 2007) could clarify the issue (c.f. Ai et al. 2007 for bee).

The projection from Jo reaches the deutocerebrum of the brain prior to mid-embryogenesis (Fig. 5a) and follows the route established earlier by the antennal pioneers (Boyan and Ehrhardt 2015). Axons target the embryonic striate, mechanosensory neuropils of the deutocerebrum (Fig. 5a) and so bypass its glomerular region (Fig. 5b) where the afferents from odor-detecting sensilla terminate (Hansson et al. 1996; Strausfeld 2009), confirming previous reports for the adult (Williams 1972; Gewecke 1979). How these embryonic mechanosensory neuropils translate into the mature AMMC with its LAMMC and MAMMC subdivisions (von Hadeln et al. 2018) remains to be established.

Axon-specific immunolabeling (Fig. 5c) reveals that the embryonic Jo axons progress medially through the brain in the so-called Tractus Pedicello-Lobus Dorsalis (TPLD; c.f. Figure 1) and contact the primary axon scaffold of the protocerebrum where they terminate as the Tractus Pedicello-Protocerebrum Posterior-medianus (TPPP) at mid-embryogenesis. At 85% of embryogenesis (Fig. 6a), these projections may be putatively allocated to the posterior slope (PS) and their terminals are morphologically very similar to those reported for the adult (Figs. 1, 6b–d).

#### Conserved tracts in the adult brain

Gewecke (1979) reports the TPPP of the adult locust to be a dense bundle comprising approximately 12 axons, and our axon counts based on Golgi (Fig. 6) and Bielschowsky (Fig. 7b) histology match his findings, implying that each of the 12 cell clusters contributes a single axon to the TPPP. Inspection of our 3D confocal data here (Fig. 3c) and previously (Boyan and Ehrhardt 2022) suggests this is the case. Despite the axons in TPLD and TPPP remaining bundled, their terminations appear to form two putative sub-fields (1, 2; Fig. 6c), but it remains to be proven whether these reflect the dorsal/ventral location of cell clusters in the pedicellar epithelium and therefore a topographic projection in the brain as demonstrated for the bee (Ai et al. 2007) and ant (Grob et al. 2021) where three terminal zones (T6I, T6III-I, T6III-II) reflect the posterior, anterior, and ventral scolopidial fields of Jo.

Gewecke (1979) hypothesized that the TPPP of the locust brain might be homologous to a tract in the bee referred to as B41 by Pareto (1972), and as T6I by Suzuki (1975). More recent findings on the bee (Ai et al. 2007) and desert ant *Cataglyphis* (Grob et al. 2021) each show subsets of fibers from Jo projecting to the protocerebrum via a branch these authors also refer to as T6I and which bear a great similarity to subfield 1 of the TPPP in the locust (c.f. Figs. 5c, 6b).

Our data show that Jo axons are found in subregions of the antennal mechanosensory and motor center (AMMC) such as LAMMC and MAMMC and also appear to target the posterior slope (PS). Pedicellar and scapal mechanoreceptors have been shown to target the MAMMC of the locust (von Hadeln et al. 2018) consistent with data from bee, ant, and fly (Kamikouchi et al. 2006; Brockmann and Robinson 2007; Patella and Wilson 2018). These parallels suggest that information from Johnston’s organ is represented in evolutionarily conserved neuropils in the insect brain.

#### Brain neuroarchitecture and behavior

Resolving the neuronal circuitry mediating Johnston’s organ-based behavior requires knowledge about the location of Jo afferents with respect to the fine neuroarchitecture of the adult locust brain. Our present study utilizes a detailed map of brain neuropils (von Hadeln et al. 2018) and a groundplan of commissures (Boyan et al. 1993) to map the terminals of these afferents to a restricted subset of mechanosensory neuropils (AMMC, PS) and commissures (nDC XVI, nDC XVII) in the brain. Molecular and cellular evidence (Boyan et al. 2003; Ito et al. 2014) confirms that these commissures are protocerebral, as deutocerebral cells choose a protocerebral commissure (nPC XX) dorsal (n, anterior) to the esophagous or a tritocerebral (TCC, nPC XXIV) ventral (n, posterior) to the esophagous to cross the midline.

Commissures nDC XVI and nDC XVII are known to contain the processes of identified multimodal interneurons (Fig. 7; O'Shea and Williams 1974; O'Shea et al. 1974) whose axons descend in the ventral nerve cord to thoracic motor regions where they regulate course corrective flight behavior (Rowell 1971, 1988; Bacon and Möhl 1979; Griss and Rowell 1986). Morphological overlap and synaptic connections between Jo afferents and such premotor interneurons have yet to be demonstrated in the locust, but outputs from wind-sensitive antennal afferents to flight motor systems via descending interneurons have been reported in *Drosophila* (Mamiya and Dickinson 2015). With both the terminals of the Jo afferents now positioned with respect to identified brain neuropils, and an identified subset of commissures containing the axons of known premotor interneurons, the locust brain offers an excellent opportunity to unravel the circuitry involved in Jo-mediated behavior. Future comparisons with the data from *Drosophila* (Mamiya and Dickinson 2015) can offer insights into evolutionary aspects of this behavior.

### Supplementary Information

Below is the link to the electronic supplementary material.

Supplementary file1 (PNG 397 kb)

High resolution image (TIF 712 KB)

## Data Availability

Core data supporting this study are available on request from Dr. E.E. Ehrhardt, AG Ito, Institute of Zoology, Universität Köln, Zülpicher Str 47b, 50,674 Cologne, Germany.

## References

[CR1] Ai H, Nishino H, Itoh T (2007). Topographic organization of sensory afferents of Johnston’s organ in the honeybee brain. J Comp Neurol.

[CR2] Anderson DT (1973). Embryology and phylogeny in annelids and arthropods.

[CR3] Bacon JP, Möhl B (1979). Activity of an identified wind interneurone in a flying locust. Nature.

[CR4] Bentley D, Keshishian H, Shankland M, Torian-Raymond A (1979). Quantitative staging of embryonic development of the grasshopper, *Schistocerca nitens*. J Embryol Exp Morphol.

[CR5] Boekhoff-Falk G (2005). Hearing in *Drosophila*: development of Johnston´s organ and emerging parallels to vertebrate ear development. Dev Dyn.

[CR6] Blöchl R, Selzer R (1988). Embryogenesis of the connective chordotonal organ in the pedicel of the American cockroach: cell lineage and morphological differentiation. Cell Tissue Res.

[CR7] Boyan G, Ehrhardt E (2015). Pioneer neurons of the antennal nervous system project to protocerebral pioneers in the grasshopper *Schistocerca gregaria*. Dev Genes Evol.

[CR8] Boyan G, Ehrhardt E (2019). Dysregulation of axogenesis in the antennal nervous system of the embryonic grasshopper *Schistocerca gregaria*. Inv Neurosci.

[CR9] Boyan G, Ehrhardt E (2022). Early embryonic development of Johnston’s Organ in the antenna of the desert locust *Schistocerca gregaria*. Dev Genes Evol.

[CR10] Boyan G, Niederleitner B (2011). Patterns of dye coupling involving serotonergic neurons provide insights into the cellular organization of a central complex lineage of the embryonic grasshopper *Schistocerca gregaria*. Dev Genes Evol.

[CR11] Boyan GS, Williams JLD (2004). Embryonic development of the sensory innervation of the antenna of the grasshopper *Schistocerca gregaria*. Arthr Struct Dev.

[CR12] Boyan GS, Williams JLD (2007). Embryonic development of a peripheral nervous system: nerve tract associated cells and pioneer neurons in the antenna of the grasshopper *Schistocerca gregaria*. Arthr Struct Dev.

[CR13] Boyan GS, Hirth F, Reichert H (2003). Commissure formation in the embryonic insect brain. Arthr Struct Dev.

[CR14] Boyan GS, Williams L, Meier T (1993). Organization of the commissural fibers in the adult brain of the locust. J Comp Neurol.

[CR15] Brockmann A, Robinson GE (2007). Central projections of sensory systems involved in honey bee dance language communication. Brain Behav Evol.

[CR16] Chang WS, Serikawa K, Allen K, Bentley D (1992). Disruption of pioneer growth cone guidance in vivo by removal of glycosylphosphatidylinositol-anchored cell surface proteins. Development.

[CR17] Dickson BJ (2002). Molecular mechanisms of axon guidance. Science.

[CR18] Eberl DF, Boekhoff-Falk G (2007). Development of Johnston’s organ in *Drosophila*. Int J Dev Biol.

[CR19] Ehrhardt E, Liu Y, Boyan GS (2015). Axogenesis in the antennal nervous system of the grasshopper *Schistocerca gregaria* revisited: the base pioneers. Dev Genes Evol.

[CR20] Ganfornina MD, Sánchez D, Bastiani MJ (1995). Lazarillo, a new GPI-linked surface lipocalin, is restricted to a subset of neurons in the grasshopper embryo. Development.

[CR21] Gewecke M (1970). Antennae: another wind-sensitive receptor in locusts. Nature.

[CR22] Gewecke M (1972). Bewegungsmechanismus und Gelenkrezeptoren der Antennen von *Locusta migratoria* L. (Insecta, Orthoptera). Zeit Morph Ökol Tiere.

[CR23] Gewecke M (1979). Central projections of antennal afferents for the flight motor in *Locusta migratoria* (Orthoptera: Acrididae). Entom Gen.

[CR24] Goodman CS (1996). Mechanisms and molecules that control growth cone guidance. Ann Rev Neurosci.

[CR25] Göpfert M, Robert D (2001). Turning the key on *Drosophila* audition. Nature.

[CR26] Göpfert M, Robert D (2001). Active auditory mechanics in mosquitoes. Proc R Soc Lond B.

[CR27] Griss C, Rowell CHF (1986). Three descending interneurons reporting deviation from course in the locust. I. Anatomy. J Comp Physiol A.

[CR28] Grob R, Tritscher C, Grübel K, Stigloher C, Groh C, Fleischmann PN, Rössler W (2021). Johnston’s organ and its central projections in Cataglyphis desert ants. J Comp Neurol.

[CR29] Hansson BS, Ochieng SA, Grosmaitre X, Anton S, Njagi PGN (1996). Physiological responses and central nervous projections of antennal olfactory neurones in the adult desert locust, *Schistocerca gregaria* (Orthoptera: Acrididae). J Comp Physiol A.

[CR30] Ho RK, Goodman CS (1982). Peripheral pathways are pioneered by an array of central and peripheral neurones in grasshopper embryos. Nature.

[CR31] Ito K, Shinomiya K, Ito M, Armstrong JD, Boyan G, Hartenstein V, Harzsch S, Heisenberg M, Homberg U, Jenett A, Keshishian H, Restifo LL, Rössler W, Simpson JH, Strausfeld NJ, Strauss R, Vosshall LB (2014). A systematic nomenclature for the insect brain. Neuron.

[CR32] Jan LY, Jan YN (1982). Antibodies to horseradish-peroxidase as specific neuronal markers in *Drosophila* and grasshopper embryos. Proc Natl Acad Sci USA.

[CR33] Jarman AP, Romand R, Varela-Nieto I (2014). Development of the auditory organ (Johnston’s organ) in *Drosophila*. Development of auditory and vestibular systems.

[CR34] Kamikouchi A, Shimada T, Ito K (2006). Comprehensive classification of the auditory sensory projections in the brain of the fruit fly *Drosophila melanogaster*. J Comp Neurol.

[CR35] Keil TA (1997). Comparative morphogenesis of sensilla: a review. Int J Insect Morphol Embryol.

[CR36] Keil TA, Steiner C (1990). Morphogenesis of the antenna of the male silk moth, *Antheraea polyphemus*. II. Differential mitoses of ‘dark’ precursor cells create the Anlagen of sensilla. Tissue Cell.

[CR37] Mamiya A, Dickinson MH (2015). Antennal mechanosensory neurons mediate wing motor reflexes in flying *Drosophila*. J Neurosci.

[CR38] Manning A (1967). Antennae and sexual receptivity in *Drosophila melanogaster* females. Science.

[CR39] O'Shea M, Williams JLD (1974). The anatomy and output connection of a locust visual interneurone; the lobula giant movement detector (LGMD) neurone. J Comp Physiol.

[CR40] O'Shea M, Rowell CHF, Williams JLD (1974). The anatomy of a locust visual interneurone; the descending contralateral movement detector. J Exp Biol.

[CR41] Patella P, Wilson RI (2018). Functional maps of mechanosensory features in the *Drosophila* brain. Current Biol.

[CR42] Pareto A (1972). Die zentrale Verteilung der Fühlerafferenz bei Arbeiterinnen der Honigbiene, *Apis mellifera* L. Z Zellforsch.

[CR43] Rowell CHF (1971). The Orthopteran Descending Movement Detector (DMD) neurones: A characterisation and review. Z Vergl Physiol.

[CR44] Rowell CHF (1988). Mechanisms of flight steering in locusts. Experientia.

[CR45] Sánchez D, Ganfornina MD, Bastiani MJ (1995). Developmental expression of the lipocalin Lazarillo and its role in axonal path finding in the grasshopper embryo. Development.

[CR46] Schmidt K (1967). Die Entwicklung der Scolopidien im Johnston’schen Organ von *Aedes aegypti* während der Puppenphase. Verh Dtsch Zool Ges.

[CR47] Seeger M, Tear G, Ferres-Marco D, Goodman CS (1993). Mutations affecting growth cone guidance in *Drosophila*: genes necessary for guidance toward or away from the midline. Neuron.

[CR48] Seidel C, Bicker G (2000). Nitric oxide and cGMP influence axogenesis of antennal pioneer neurons. Development.

[CR49] Singhania A, Grueber WB (2014). Development of the embryonic and larval peripheral nervous system of *Drosophila*. Wiley Interdiscip Rev Dev Biol.

[CR50] Strausfeld NJ (1976). Atlas of an insect brain.

[CR51] Strausfeld NJ (2009). Brain organization and the origin of insects: an assessment. Proc R Soc B.

[CR52] Suzuki H (1975). Antennal movements induced by odour and central projection of the antennal neurones in the honey-bee. J Insect Physiol.

[CR53] Tischner H (1953). Über den Gehörsinn von Stechmücken. Acustica.

[CR54] Todi SV, Sharma Y, Eberl DF (2004). Anatomical and molecular design of the *Drosophila* antenna as a flagellar auditory organ. Microsc Res Tech.

[CR55] von Hadeln J, Althaus V, Häger L, Homberg U (2018). Anatomical organization of the cerebrum of the desert locust *Schistocerca gregaria*. Cell Tissue Res.

[CR56] Williams JLD (1972) Some observations on the neuronal organization of the supraoesophogeal ganglion in *Schistocerca gregaria*, Forskål, with particular reference to the central complex. Ph.D. Thesis, University of Wales, Cardiff

[CR57] Williams JLD (1975). Anatomical studies of the insect central nervous system: a ground-plan of the midbrain and an introduction to the central complex in the locust, *Schistocerca gregaria* (Orthoptera). J Zool Lond.

