# Supplementary figures and images for: Central projections from Johnston’s organ in the locust: Axogenesis and brain neuroarchitecture
Source: Dev Genes Evol. 2023 Sep 11;233(2):147–59. doi: 10.1007/s00427-023-00710-0 (PMC10746777; doi:10.1007/s00427-023-00710-0)

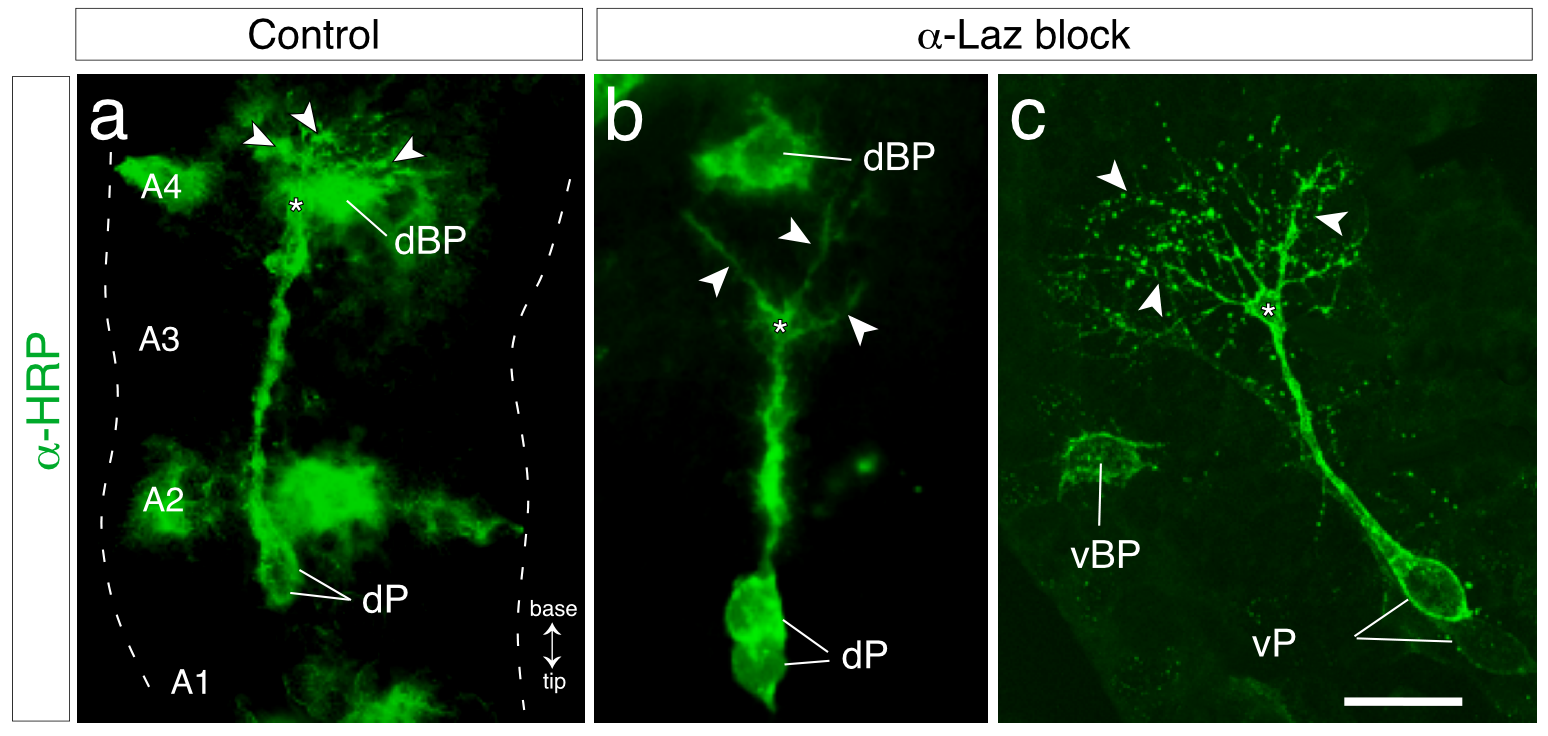

Supplement: Supplementary file 1 — Supplementary file1 (PNG 397 kb) [file 427_2023_710_Fig8_ESM.png]

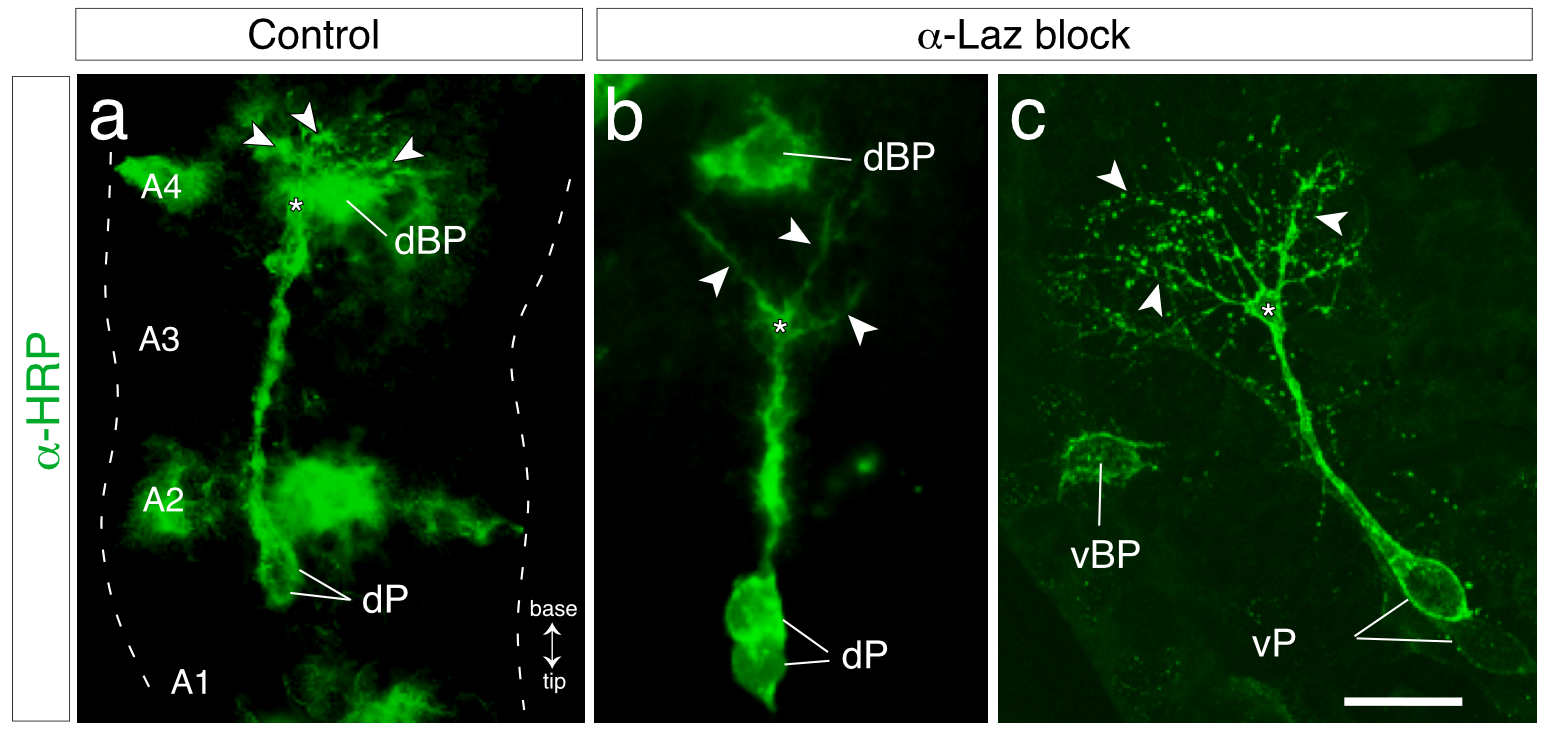

Supplement: Supplementary file 2 — High resolution image (TIF 712 KB) [file 427_2023_710_MOESM1_ESM.tif]
